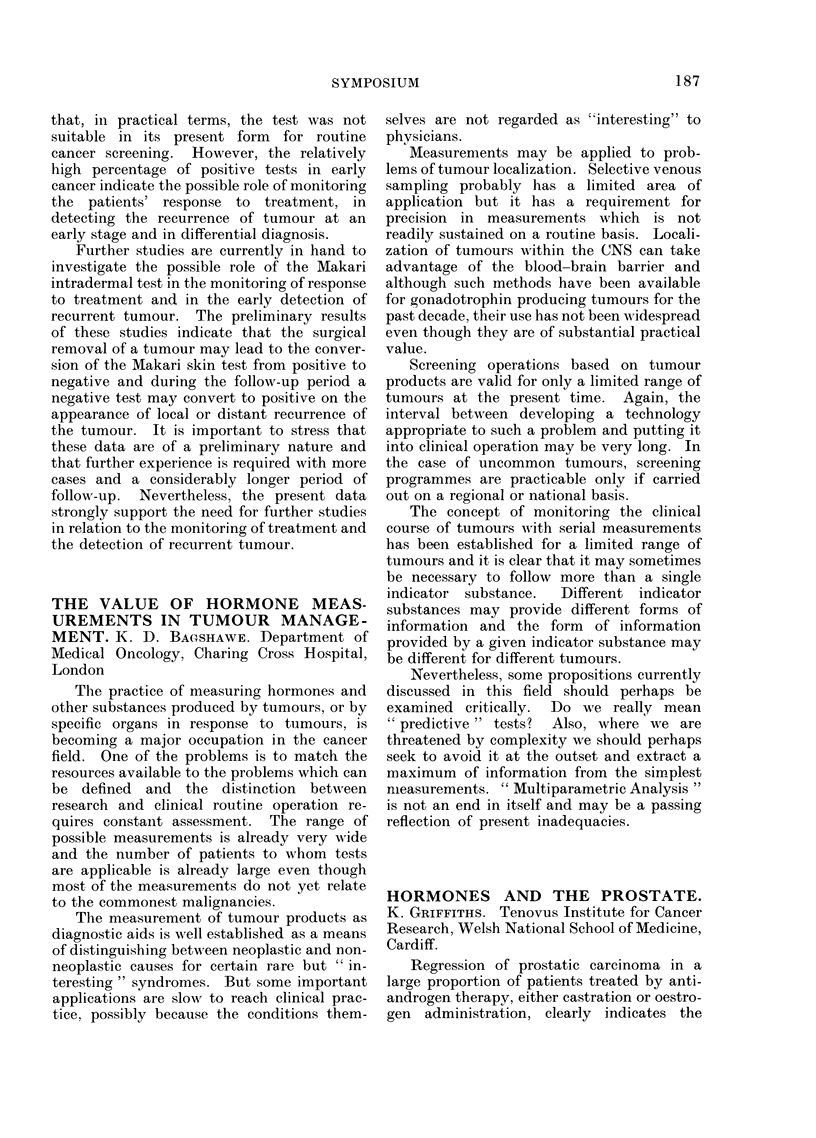# Proceedings: The value of hormone measurements in tumour management.

**DOI:** 10.1038/bjc.1974.175

**Published:** 1974-08

**Authors:** K. D. Bagshawe


**THE VALUE OF HORMONE MEASUREMENTS IN TUMOUR MANAGEMENT.** K. D. Bagshawe. Department of Medical Oncology, Charing Cross Hospital, London

The practice of measuring hormones and other substances produced by tumours, or by specific organs in response to tumours, is becoming a major occupation in the cancer field. One of the problems is to match the resources available to the problems which can be defined and the distinction between research and clinical routine operation requires constant assessment. The range of possible measurements is already very wide and the number of patients to whom tests are applicable is already large even though most of the measurements do not yet relate to the commonest malignancies.

The measurement of tumour products as diagnostic aids is well established as a means of distinguishing between neoplastic and non-neoplastic causes for certain rare but " interesting " syndromes. But some important applications are slow to reach clinical practice, possibly because the conditions themselves are not regarded as "interesting" to physicians.

Measurements may be applied to problems of tumour localization. Selective venous sampling probably has a limited area of application but it has a requirement for precision in measurements which is not readily sustained on a routine basis. Localization of tumours within the CNS can take advantage of the blood–brain barrier and although such methods have been available for gonadotrophin producing tumours for the past decade, their use has not been widespread even though they are of substantial practical value.

Screening operations based on tumour products are valid for only a limited range of tumours at the present time. Again, the interval between developing a technology appropriate to such a problem and putting it into clinical operation may be very long. In the case of uncommon tumours, screening programmes are practicable only if carried out on a regional or national basis.

The concept of monitoring the clinical course of tumours with serial measurements has been established for a limited range of tumours and it is clear that it may sometimes be necessary to follow more than a single indicator substance. Different indicator substances may provide different forms of information and the form of information provided by a given indicator substance may be different for different tumours.

Nevertheless, some propositions currently discussed in this field should perhaps be examined critically. Do we really mean " predictive " tests? Also, where we are threatened by complexity we should perhaps seek to avoid it at the outset and extract a maximum of information from the simplest measurements. " Multiparametric Analysis " is not an end in itself and may be a passing reflection of present inadequacies.